# Cyberloafing: Exploring the Role of Psychological Wellbeing and Social Media Learning

**DOI:** 10.3390/bs13080649

**Published:** 2023-08-03

**Authors:** Shwetha M. Krishna, Somya Agrawal

**Affiliations:** 1 HR, OB and Communications Area, T A Pai Management Institute, Manipal Academy of Higher Education, Manipal 576104, India; shwetha.mk@manipal.edu; 2 Department of Information Management, Chaoyang University of Technology, Taichung 413310, Taiwan

**Keywords:** cyberloafing, social media learning, psychological wellbeing, postsecondary education

## Abstract

Due to the advances in internet communications technology (ICT), the use of digital devices, such as laptops, tablets, or smartphones, in the educational setting has become very common among young people. A considerable body of research has shown that there are adverse effects of in-class internet usage, termed “cyberloafing” on students’ academic performance, making it a rising concern for scholars. Within this context, the present study examines cyberloafing as a multidimensional construct and studies the mediating effects of psychological wellbeing and social media learning between cyberloafing behaviour and cyberloafing activities of students. Using an online survey, data was collected from 240 undergraduate and graduate students at a private university in India. The data were analyzed using structural equation modelling and mediation analysis. The results indicate that cyberloafing behaviour negatively influences student’s psychological wellbeing, whereas psychological wellbeing is positively related to cyberloafing activities. It was also found that, on one hand, cyberloafing behaviour negatively influences social media learning, whereas social media learning did not have any effect on cyberloafing activities in students. This study highlights that it is crucial for educators and course instructors to incorporate appropriate practices and interventions to manage the misuse of the internet through cyberloafing in classrooms.

## 1. Introduction

Rapid advancements in information and communication technologies (ICT) have diminished the time and space constraints of using the internet, as individuals can enjoy the internet at any time and place using a handheld device [1,2,3]. This has led to an increase in the dependence on internet devices among the youth, especially in the last two decades [4], making it a problematic concern for scholars. Ideally, course facilitators require that, during classes, students use digital devices in the right manner [5], i.e., primarily for class-related tasks such as looking for information pertaining to their lectures, during in-class assignments, or participating in class quizzes online, to encourage learning. However, studies have found that students increasingly use the internet for personal interests during classes [6]. Students use technology mostly for socialization, followed by news reading and personal business [7]. They prefer engaging in web-based activities on various social media networking sites and using other programs during classes [8,9] rather than focusing on learning and studying. Dursun et al. [10], in their mixed method survey with a sample of 1854 students, demonstrated that increased use of technology and a negative attitude towards class increases cyberloafing, and cyberloafing is associated with higher amounts of time spent on activities such as sharing, shopping, real-time updating, and accessing content online. This behaviour is called cyberloafing [11].

Cyberloafing has generally been used as a one-dimensional construct, describing the various activities an individual indulges in during cyberloafing. Interestingly, findings on cyberloafing have shown both positive and negative consequences. Regarding it as a multidimensional construct rather than a one-dimensional construct [12] may explain the contradicting consequences of cyberloafing. Therefore, in this research, cyberloafing is approached as a multidimensional construct (cyberloafing behaviours and cyberloafing activities) which has not received much attention in past research. Scholars have primarily examined the antecedents and outcomes of individual differences in cyberloafing [3]. An attempt has often been made to find various internal and external constructs that cause this behaviour [1]. However, not enough research exists which elaborates on the division and the order of cyberloafing into the constructs of behaviour and activities, where one acts as the antecedent of the other [13,14].

The use of emerging mobile technologies is raising the levels of cyberloafing among university students. Within this context, the present study adds to the existing literature about cyberloafing within higher education by examining the interrelationship between cyberloafing behaviour and activities among university students. It extends the previous research by providing insightful clues on how cyberloafing behaviours impact the psychological wellbeing and learning through social media in students. The results of the consequences of cyberloafing are contradictory, making it difficult to determine the appropriate actions. Therefore, by providing an overview of the consequences and offering possible ways of intervention, both theory and practice can be helped, offering interesting opportunities for further research. Theoretically, this study provides further empirical evidence that the multidimensional construct of cyberloafing is valid and the prevalence of cyberloafing in this study proves that all the activities and behaviours of cyberloafing are present within the classroom environment. Practically, to change students’ behaviour, course facilitators should understand why students are engaging in cyberloafing so they can perform interventions to either diminish the negative effects or utilize the positive effects of cyberloafing.

Developed in the area of Communication Studies [15], the uses and gratifications (U & G) theory states that people choose and consume particular media to obtain satisfaction from having interests, needs, or goals fulfilled [16]. For example, maintaining relationships is considered as one of the dimensions of the U & G theory, where internet-based email is often used to communicate with family and friends [17]. Sometimes, personal content is included in email messages, and people find such a way of sharing to be gratifying [18]. Therefore, past research states that there is a link between students’ internet use and their psychological wellbeing [19,20]. However, Becker et al. [20] found that using the internet inside the classroom is linked to lower levels of emotional wellbeing, increased depressive symptoms, and social anxiety. A higher level of cyberloafing behaviour leads to lower psychological wellbeing, but this relationship may not be strictly linear. The nonlinear effect of cyberloafing on psychological wellbeing may result from frequency, duration, and the extent to which it interferes with work responsibilities. Studies investigating the correlation between cyberloafing and wellbeing indicate that the impact of cyberloafing on psychological wellbeing cannot be categorized as universally positive or negative. Furthermore, the perceived advantages and disadvantages of cyberloafing are not definitive indicators of its overall extent.

Enhanced use of technology in educational settings increases the chances of a student’s exposure to distractions [21], leading to off-task activity and multitasking [22,23]. Studies show that off-task internet usage not only affects a student’s own learning but also affects their neighboring students’ learning [24]. As revealed in a survey of 1445 students from three Southern African countries conducted by le Roux et al. [25], multitasking leads to lower academic performance. Similarly, Dönmez and Akbulut [26], in their experimental study, reported that multitasking is related to weaker learning gains. On the other hand, Cerretani et al. [27] demonstrated that a lack of or extensive use of technology was associated with increased distress, more difficulties, and poor general functioning. Excessive and problematic internet usage can also lead to extreme and unhealthy behaviour of smartphone addiction [28], and decrease academic performance [29]. Considering the above-mixed results, there is still a lack of empirical research on the psychological effects of inside-classroom internet use. Moreover, it would be intriguing to examine whether such cyberloafing behaviours could further impact the psychological wellbeing of students by instigating students to indulge in cyberloafing activities such as ‘searching for online support’. The existing literature is unable to give enough empirical evidence in this regard.

Previous studies [30,31] have found that students also use various social media platforms while completing class-related activities. When students feel demotivated to complete assignments by themselves and tend to postpone school activities, SNSs present opportunities for interaction and learning through instant messaging, simultaneous chat, and tracking of class members [32]. Students can complete their classwork and assignments faster using discussion forums, YouTube videos, etc., compared to other students who incorporate purely self-based learning [33]. Hence, these environments consist of various tools for organizing and exchanging information and synergistic aspects of the learning experience. Using social media platforms in the teaching process increases the academic performance of students, which facilitates learning, improves communication, and increases the student’s motivation and positive attitudes toward courses [34]. Therefore, including social media in education could also have many positive reflections on educational environments at all levels of education [35]. Going further, using them during different time periods of classroom teaching could facilitate learning among students [34]. From this perspective, cyberloafing activities could also be mitigated to a certain level. Therefore, the final objective of this study is to examine whether cyberloafing behaviour impacts learning through social media, which further instigates students to engage in cyberloafing activities.

To summarize, the research questions of this study are as follows:

1. Does cyberloafing behaviour in students instigate them to engage in cyberloafing activities in class?
2. Does cyberloafing behaviour influence psychological wellbeing, which instigates students to engage in cyberloafing activities?
3. Does cyberloafing behaviour impact learning through social media, which further instigates students to engage in cyberloafing activities?

## 2. Hypotheses and Literature Review

**Figure 1:**
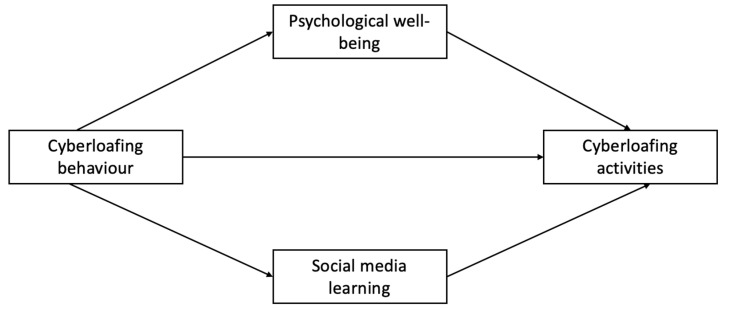
Research framework.

This section presents the literature on all the variables. The hypothesized research model is depicted in Figure 1. Cyberloafing behaviour is the independent variable, and cyberloafing activities are the dependent variable. Psychological wellbeing and social media learning are the mediating variables. Through this hypothesized model, the present study examines the mediating effects of psychological wellbeing and social media learning in the relationship between cyberloafing behaviour and cyberloafing activities of students in higher education.

### 2.1. Psychological WellBeing and Cyberloafing Behaviour

Cyberloafing is defined as “the use of the Internet and information technologies tools at work/school environment by individuals for personal purposes during work/school hours)” [11]. Through investigations on the different facets of cyberloafing in the workplace, Doorn [12] established that cyberloafing has a multidimensional structure. Past research has mainly emphasized cyberloafing activities. This study included the behaviours of cyberloafing with the objective of clarifying the reasons why students engaged in cyberloafing. Doorn [12] explains four main cyberloafing behaviours in his research, such as development, recovery, deviant, and addictive behaviours. Developmental behaviour uses the process of cyberloafing as a potential source of learning. This perspective can provide an increase of skills which could be used in future activities by individuals [36]. Recovery behaviour is considered from the health perspective of the individual. The deviant behaviour considers cyberloafing as an unwanted behaviour aimed against the context in which it is used. It is considered a behaviour with negative consequences (decreased productivity) for individuals [37,38]. Lastly, the addictive behaviour could be caused by engaging in cyberloafing as a habit and could result in problems. In this study, we have focused on the deviant and development aspects of cyberloafing behaviour to examine the possibility of both the positive and negative consequences of cyberloafing.

In the field of education, studies have linked cyberloafing within the classroom environment with the use of information communication technologies [39,40,41]. Some researchers highlighted the benefits of using smart devices inside the classroom, such as heightened participation, more interactions with the course facilitator, and active learning [42,43]. In contrast, others highlighted drawbacks, such as decreased attention, missing instruction and lecture notes, lower grades among students [1], and poor academic performance [44,45,46]. Therefore, cyberloafing behaviour can be classified in terms of its outcomes, i.e., positive or negative. Positive outcomes consist of cyberloafing being used for development, i.e., as a resource for upgrading skills and learning [36] and for recuperation, i.e., as a technique of mitigating uneasiness and positively affecting individual health [13,47]. On the contrary, negative outcomes consist of deviant behaviour, which diminishes productivity [37] and increases addictive behaviour [38] characterized as habitual and difficult to cope with [7].

Previous studies [1,19,48,49] have demonstrated a link between students’ internet use and their psychological wellbeing. For example, both cross-sectional and longitudinal studies examining the interrelationship between the use of social networking sites (SNSs) and psychological wellbeing among students have demonstrated that SNS use is negatively associated with students’ overall wellbeing [48,50,51], revealing further associations between media multitasking and lower emotional wellbeing indicated by depressive symptoms and higher social anxiety [20]. Rosen et al. [52] and van Der Schuur et al. [53] particularly emphasized that media-instigated task switching and low cognitive control over switching behaviour hinder students’ performance and wellbeing [52]. Researchers have underlined that task sidelining and procrastination with social media leads to academic stress and SNS-instigated strains impeding students’ academic and overall wellbeing [48,50]. They also found that frequent checking of social media accounts is related to loneliness [19] and spending more time scrolling on social media platforms was associated with increased anxiety [49], depression [52], and reduced satisfaction with life [51]. Thus, based on the findings in the above literature, the following hypothesis was generated.

**Hypothesis** **1** **(H1).** *Cyberloafing behaviour is significantly related to the psychological wellbeing of students.*

### 2.2. Cyberloafing Activities and Psychological WellBeing

Li and Chung [54] distinguished cyberloafing activities into four types: social (e.g., Facebook, Instagram, Twitter) and sharing (e.g., Blogger); informational (e.g., internet searches); for leisure purposes (e.g., downloading music, playing online games, downloading software); and virtual emotional activity (e.g., dating sites, shopping online, and other unclassifiable activities). This classification is based on the functions of cyberloafing. In another study, Lim and Chen [55] divided cyberloafing into two types: browsing activities and emailing activities. This categorization is based on the level of control over the activities as well as the effort and energy requirements of the activities [12]. For this study, we included four cyberloafing activities: Social, Informational, Leisure, and Virtual Emotional. It is possible to express oneself or share information using blogs as part of social activities (e.g., Facebook). The second activity, Informational, entailed seeking information and news (e.g., CNN). Playing games online or downloading music are leisure activities (e.g., YouTube). Lastly, the Virtual Emotional activity included all internet activities that did not fit into the other three categories (e.g., shopping online). 

The conservation of resource theory (COR), posited by Dr. Hobfoll in 1989 [56], proposes that people are compelled to safeguard their energy supplies and, when they are unable to recuperate, they experience stress. In their effort–recovery model (ERM), Meijman and Mulder [57] proposed that individuals could return to full capability after taking intermittent pauses to decrease duration of work time. However, excessive work stretches may hinder an individual’s ability to recover fully. Based on COR and ERM, it is expected that a reasonable amount of cyberloafing may assist the recovery experience of students by restoring resources used up while performing academic tasks [41]. It was found that internet usage that decreases negativity and increases pleasure was higher for those with greater signs of anxiety, which, again, might result in greater susceptibility to SNS-related addiction [58]. Also, individuals usually interact with others to maintain and generate social relationships through web-based platforms known as social networking sites (SNSs) [59]. When the face-to-face social requirements are not met, such as the urge to belong, to be viewed as socially competent, and to be forceful in communication, may promote problematic SNS use, according to Casale and Fioravanti [60]. Thus, based on the findings in the above literature, the following hypothesis was generated.

**Hypothesis** **2** **(H2).** *Psychological wellbeing is significantly related to cyberloafing activities in students.*

### 2.3. Cyberloafing Behaviour and Cyberloafing Activities

Cyberloafing behaviour represents the reasons why individuals visit certain websites and is distinct from activities due to its expected positive and negative consequences. For example, individuals engage in cyberloafing activities because they want to distance themselves from the task (deviant behaviour) or are addicted to certain websites (addiction behaviour). Task requirements could influence cyberloafing from a work perspective such that high demands combined with low resources could lead to situations where cyberloafing activities can instigate cyberloafing behaviour for recovery. Therefore, cyberloafing activities and cyberloafing behaviours are distinct from each other. Additionally, the distinction between personal and student life can play a role regarding the use of the internet for private purposes and more interference between both lives could result in more cyberloafing. This study will examine the interrelationship between the activities and behaviours. This will provide insights into how activities are related to behaviours. The relation between activities and behaviours will also explain why cyberloafing is a multidimensional construct.

The combination of constructs aims to combine the action with the student’s mindset. Past research [12] shows that cyberloafing represents a combination of a cyberloafing activity with one or more behaviours. For instance, the found relations between cyberloafing activities and behaviour showed that leisure activities were related only to deviant behaviour. However, the social and informational cyberloafing activities showcased connections with all four behaviours. In other words, leisure activity only led to deviant behaviour, while social and informational activities resulted from one or even a combination of behaviours. Interestingly, cyberloafing can be used for recovery purposes when students are either emotionally or physically exhausted. In other words, certain cyberloafing behaviours confirm the recovery potential of engaging in such activities.

It has been established that the development dimension of cyberloafing behaviour is not one of the factors that influence students’ decisions to engage in cyberloafing activities [6]; however, studies by Brubaker [61] and Kalaycı [62] have contended that individuals engage in cyberloafing activities due to deviant behaviour in classes. Yaşar and Yurdugül [63] also confirmed these findings and mentioned that students showcasing deviant behaviour tend to take advantage of internet access in class settings for nonwork-related things (i.e., personal) rather than for educational reasons. They engage in activities such as playing online games, shopping online, or surfing on social media websites such as Facebook and Twitter. Additionally, Saritepeci [64] emphasizes that unauthorized access to a school’s network significantly impacts student cyberloafing behaviour. In keeping with this, we contend that there is an interrelation between internal constructs (i.e., salvation behaviour) that justify students in higher education engaging in cyberloafing activities at school. Thus, based on the findings in the above literature, the following hypothesis was generated.

**Hypothesis** **3** **(H3).** *Cyberloafing behaviour is significantly related to cyberloafing activities in students.*

### 2.4. Cyberloafing Behaviour and Social Media Learning

Mobile internet has become an essential aspect of most college students’ education and lives. Generation Z has different expectations from previous generations and has different learning preferences [65]. Here, social media learning is defined as students’ perception of using social media platforms for the exchange of information, sharing, discussing, and searching functions of social media for learning purposes [66,67]. Tang et al. [68] showed that students in Hongkong made use of the sharing, discussing, and searching functions of social media while easily getting distracted at the same time by the entertainment-related functions available. Interestingly, Lau [66] assessed 348 undergraduate students’ social media usage for learning and the study demonstrated that social media usage for learning had no effect on the academic performance of students. 

On the contrary, Šerić [69] researched college students from three different European nations and examined to what extent students used social media for learning purposes. She found a link between low usage of social media among students and professors and low perceived usefulness of social media used as a means of learning. Similar to this, Everson et al. [70] investigated the use of Facebook, Twitter, and YouTube as educational tools among graduate students and discovered that students were less willing to use social networking sites during a course than expected, as only a small number of them wanted to create and post on YouTube a brief video in order to teach their peers about topics covered in class. Gregory et al. [67] showed that Facebook could be used as an educational network by setting up a group on Facebook just for discussing math course material outside of class. This was found to improve significantly undergraduate students’ involvement, contentment, and performance in a calculus course.

The above studies show that social media has grown to be an effective tool for fostering relationships between students and their teachers, as well as with their peers, and including them in the new distance learning environment. They are adept at navigating cyberspace and are likely to utilize social media and access the internet on a regular basis [71]. Students may not even think of cyberloafing in class as problematic [41,72]. Although using various social media is useful for interactive learning and boosting student engagement, its actual use is constrained by several difficulties [73]. During class, students typically engage in web-based activities on popular social networking sites and access other programs. Students cyberloaf more for real-time updating, gaming, and accessing online content rather than spending time learning and taking notes [39]. Their interest and active engagement in classroom learning activities are reduced when they indulge in cyberloafing during class. Thus, based on the findings in the above literature, the following hypothesis was generated.

**Hypothesis** **4** **(H4).** *Cyberloafing behaviour is significantly related to learning through social media in students.*

### 2.5. Social Media Learning and Cyberloafing Activities

Students are prone to using technology inefficiently rather than for professional or academic advancement [74]. Even when social media is utilized in the classroom for learning, students may interpret it differently from the teacher. Since social media is primarily designed as a networking tool [75,76], it may enhance off-topic or nonacademic debate. Social media use among students has an inverse relationship with how much time they spend studying [77]. Using social media for learning can result in multitasking [66]. Social media usage in the classroom can distract students from the task at hand [78]. Empirical evidence demonstrates that students engage in cyberloafing activities during courses [79]. Instead of taking notes, checking prior assignments, searching for material, using multimedia, and creating presentations during class, students may use social media to play video games, exchange instant messages, listen to music, and watch videos [61]. Thus, based on the findings in the above literature, the following hypothesis was generated.

**Hypothesis** **5** **(H5).** *Learning through social media learning is significantly related to cyberloafing activities in students.*

### 2.6. Mediating Effects of Psychological Wellbeing and Social Media Learning

According to some studies [13,80], cyberloafing or personal use of the internet for nonwork-related purposes has many benefits for the wellbeing of individuals, such as improved coping with job stress and personal problems, and increased performance, creativity, job satisfaction, and productivity [3]. Vitak et al. [81] suggested that cyberloafing behaviour may be advantageous for enhancing creativity, reducing stress, increasing satisfaction, and improving psychological wellbeing. According to Coker [82], disrupting the activity, in contrast to planned breaks, increases productivity by improving the individual’s concentration. As a result, cyberloafing practices may influence a student’s performance in school settings and their personal and intellectual growth, which may lead to academic achievement [34]. According to Wu et al. [83], students may find that cyberloafing is an effective technique to re-establish their cognitive capacities. Researchers have questioned the generally held belief that cyberloafing is always harmful, stating that it can help people recover faster [82] and be more involved in their subsequent work [84]. 

Research on social media in education suggests that including social media in learning and teaching contexts may result in new kinds of inquiry, communication, cooperation, identity work, or good cognitive, social, and emotional effects [85,86]. Still, only a few students use social media in sophisticated ways that teachers could find useful [87]. Social media frequently portrays an idealised or false view of activity, one that is frequently more fascinating or enjoyable than attending class, studying, or doing coursework. Social media has relatively little educational value because it was not developed for educational purposes and students use it more as a medium for social interchange than for learning [88,89]. It has been often observed that when they have access to social media during classes, students use it for course content purposes but also for sending/receiving e-mails, surfing news and sports websites, downloading music, chatting, playing online games, reading blogs, visiting social networks and updating personal websites [79]. Kay et al. [90] evaluated the prevalence and impact of distracting behaviours when students bring their own devices to class, as well as the specifics of demographic data. According to the report, 80% of students participate in some kind of cyberloafing activity. 

Conservation of resource theory states that people always strive to retain, protect and build valued resources, and actual or potential resource loss would cause stress [56]. A resource is anything that helps individuals to attain goals [91]. People invest in resources either to avoid resource loss or to seek resource gain [92,93]. Their decision to invest in resources depends on the return on their resource invested. In this study, psychological wellbeing and social media learning can be considered resources that can help students fulfil their needs. Cyberloafing has both a negative and positive aspect, as it can cause both resource loss and resource recovery. On one hand, cyberloafing is frequently viewed as a deviant behaviour that depletes limited resources by reducing productivity or creating additional liabilities. On the contrary, it might provide an unanticipated advantage by allowing students to recharge through temporary separation from class work. The effect of cyberloafing on cyberloafing activity depends on whether students are investing their resources, i.e., psychological wellbeing and social media learning to avoid resource loss or to seek resource gain. Therefore, based on the conservation of resource theory, we propose that psychological wellbeing and social media learning mediate the relationship between cyberloafing behaviour and cyberloafing activity. The following hypotheses were generated.

**Hypothesis** **6** **(H6).** *Psychological wellbeing mediates the relationship between cyberloafing behaviour and cyberloafing activities in students.*

**Hypothesis** **7** **(H7).** *Learning through social media mediates the relationship between cyberloafing behaviour and cyberloafing activities.*

## 3. Research Methodology 

### 3.1. Participants and Data Collection Procedure

The data for this study was collected one time from undergraduate students using an online survey. The respondents were selected by using a convenience sampling technique. Convenience sampling, a nonprobability sampling strategy, involves selecting participants based on their easy accessibility in terms of location, availability, cost, time, and willingness to participate [94]. This study chose the convenience sampling strategy due to its ability to readily access the student population, its cost-effectiveness, and the voluntary nature of participation. This was done to ensure a higher response rate and to better understand cyberloafing behaviour across students from many streams rather than just one. The students registered for corporate governance, entrepreneurship development, human resource management, and organizational behaviour courses in the fall semester of 2021 at a large private university in India. To reduce the possible impact of socially desired responses on data quality, our surveys were sent to participants online through Google Forms (did not contain any personal information) to maintain high degrees of anonymity and more dependability in gathering sensitive information. They were given a brief overview of the purpose of the study and ensured anonymous responses before distributing the questionnaire. Three hundred fifty online questionnaires were distributed, of which 254 completed responses were received. Responses from 14 students were removed due to missing information. A total of 240 valid responses were considered, with a response rate of 68.5%. Due to the English proficiency of the students, the questionnaires were prepared and distributed in the English language. The data set was evaluated for missing data and sampling size. It was determined that the data set had no missing values. 

#### Symmetry and Kurtosis

To meet the criteria for sampling size assumption, the observation number to parameter number ratio must be at least 10:1 [95]. The current study satisfied this requirement. A normal distribution test of the variables showed that all skewness coefficients of the four variables were between −0.6 and +2.17 and kurtosis coefficients were between −2.09 to 0.06. The skewness and kurtosis values fall between normal ranges, i.e., ±2 for skewness and ±7 for kurtosis [95], with a slight deviation of the skewness of cyberloafing activity (2.170), which indicates data are normally distributed. The final data included 124 male students and 116 female students, as shown in Table 1. The students’ ages ranged from 17 to 26 years, with an average of 18.9. Most of the students were from the departments of engineering (*n* = 142), followed by law (*n* = 66) and management (*n* = 32).

### 3.2. Variables and Measures

This section describes the measures used for each construct in detail, along with their Cronbach’s alpha value. All the items for variables, such as cyberloafing behaviour, psychological wellbeing, social media learning, and cyberloafing activities, were rated using the Likert-type five-point scale, with ‘1’ indicating ‘strongly agree’ and ‘5’ indicating ‘strongly disagree’ in the questionnaire items. Previous studies demonstrate good psychometric properties for all the constructs. Please refer to Table A1 in Appendix A for the measurement items.

#### 3.2.1. Cyberloafing Behaviour

The variable of cyberloafing behaviour was measured using four items adapted from the scale developed by Doorn [12]. All items were measured on a five-point Likert scale ranging from one (never) to five (always). The sample items include “Avoid school tasks” and “Avoid thinking of work tasks”. The Cronbach alpha value for this variable showcases good reliability with α = 0.816.

#### 3.2.2. Psychological Wellbeing

Psychological wellbeing was measured using a five-item scale adopted from [96]. The sample items include “I have been feeling cheerful” and “I have been feeling good about myself”. All items were measured on a five-point Likert scale ranging from one (none of the time) to five (all the time). The Cronbach alpha value for this variable showcases good reliability with α = 0.872.

#### 3.2.3. Social Media Learning

This variable was measured using four items adopted from Mills et al. [97]. The scale specifically assessed how university students felt about using social media for online community learning and class participation. Sample items include “Posting questions to my classmates/friends helps me understand my readings better” and “I am able to get faster feedback from my peers”. The items were rated on a five-point Likert-type scale from one (strongly disagree) to five (strongly agree). The Cronbach alpha value for this variable showcases good reliability with α = 0.78.

#### 3.2.4. Cyberloafing Activities

The variable of cyberloafing activities was measured using six items adapted from Doorn [12]. Students were instructed to rate on a five-point scale how frequently they engage in web-based activities during the class. Sample items include “Shop online” and “Express my opinion—Twitter/LinkedIn”. All items were measured on a five-point Likert scale ranging from one (Never) to five (Always). The Cronbach alpha value for this variable showcases good reliability with α = 0.728.

#### 3.2.5. Common Method Variance

To test for the common method bias, we conducted an exploratory factor analysis using Harman’s single-factor test [98]. After extracting a single factor, the test revealed that the single factor explained a total variance of 17.985%, which does not exceed the commonly accepted threshold of 50%. This suggests that common method bias is not a problem with this dataset.

## 4. Data Analysis and Results

Data analysis was carried out using multiple steps. First, we presented descriptive statistics showcasing the correlation values for the measured variables. Thereafter, we carried out structural equation modeling (SEM) to define a theoretical causal model consisting of a set of predicted covariances between variables and then tested whether it is plausible when compared to the observed data [99,100]. The fit of the proposed model to the data was estimated using SEM analysis based on the measurement model. Several widely known model fit adequacy indices, including an χ^2^, standardized root mean square residual (SRMR), a goodness-of-fit index (GFI), the comparative fit index (CFI), the incremental fit index (IFI), and the root mean square error of approximation, were employed to assess model fit (RMSEA). These model fit indices indicate how much a research model outperforms a null or independent model in terms of overall fit [95]. To examine the mediation effects, we used the Sobel test [101] and a bootstrapping approach (bootstrap = 5000) [102], as presented in Section 4.4.

The following section presents the results of the data analysis. 

### 4.1. Descriptive Statistics

Table 2 presents the descriptive statistics and Pearson correlation values for the measured variables. The mean score for social media learning is higher than other variables. The mean score for cyberloafing activities (3.60), and cyberloafing behaviour was lower than average (2.31 and 2.47, respectively). The mean score of psychological wellbeing is higher than average (3.44). The construct of cyberloafing activities shows positive correlations with cyberloafing behaviour, psychological wellbeing, and social media learning. Similarly, psychological wellbeing is positively correlated with social media learning. The results also show a negative correlation between cyberloafing behaviour and psychological wellbeing. However, the correlation between social media learning and cyberloafing behaviour is negative.

### 4.2. Measurement Model

In Table 3, we summarize all the model-fit indices. The table confirms that the model-fit indexes of the measurement model (χ^2^/DF = 1.664, *p* ≤ 0.001; CFI = 0.938, GFI = 0.904, SRMR = 0.0638, and RMSEA = 0.053) justify that further examination of the structural model is needed.

### 4.3. Structural Model

**Figure 2:**
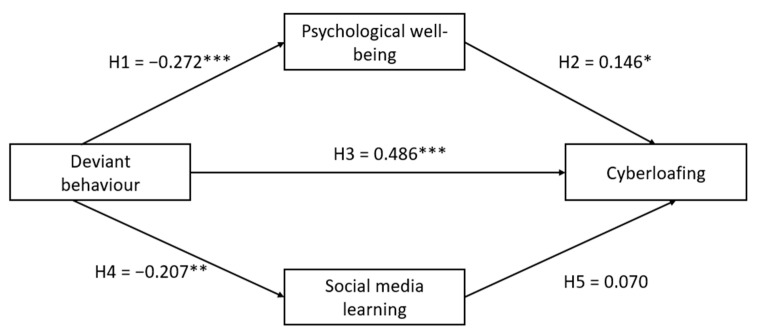
SEM model with results of mediation analysis. Note: * *p* < 0.05; ** *p* < 0.01; *** *p* ≤ 0.001 (two-tailed test).

Figure 2 shows the overall structural model with the path coefficients. The results showed that the hypothesized model fits the data well (χ^2^/DF = 1.716, *p* ≤ 0.001; CFI = 0.932, GFI = 0.900, SRMR = 0.0803, RMSEA = 0.055). According to Hu and Bentler [103] an SRMR value that lies between the range of 0 and 0.08 is acceptable. 

**Hypothesis 1** states that cyberloafing behaviour is significantly related to psychological wellbeing. We found support for it (β = −0.272, *p* ≤ 0.001). **Hypothesis 2**, which states that psychological wellbeing is positively related to cyberloafing activities, found significant but partial support (β = 0.146, *p* ≤ 0.05). **Hypothesis 3** tested that there is a significant relationship between cyberloafing behaviour and cyberloafing activities. This hypothesis found significant support with (β = 0.486, *p* ≤ 0.001). **Hypothesis 4** states that cyberloafing behaviour is significantly related to social media learning. The results for this hypothesis were significant and negative (β = −0.207, *p* ≤ 0.01). In **Hypothesis 5**, the relationship between social media learning and cyberloafing activities was tested. The results were not significant (β = 0. 070, *p* ≥ 0.01). Therefore, Hypothesis 5 was rejected. 

### 4.4. Mediation Analysis

**Figure 3:**
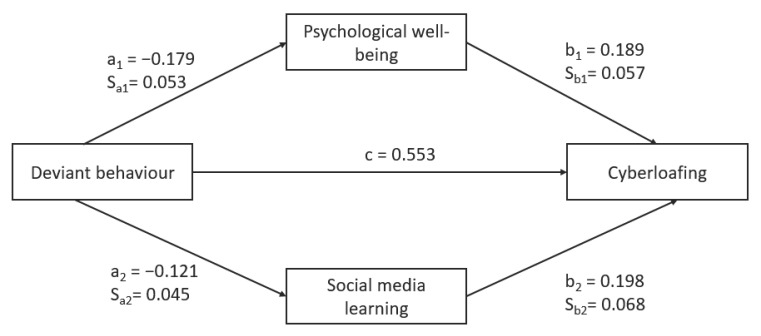
Unstandardized Regression Coefficients and Standard Errors.

Next, we carried out a mediation analysis by following the works of Sobel [101] and Preacher [104]. In the past, several statistical techniques [95,101,105] have been used to examine the effect of a mediating variable on independent and dependent variables. The previous paragraph presented the results of structural equation modeling (SEM). To carry out further analysis, we made use of the Sobel test and Hayes SPSS Process Macro to validate our results for mediation analysis. There are two primary methods for formally testing the significance of the indirect test. Figure 3 shows that our research model tested two mediation effects. 

### 4.5. Sobel Test

The Sobel test [101] was utilized to examine the two mediation models. In the first mediation effect, we tested whether psychological wellbeing mediates the relationship between cyberloafing behaviour and cyberloafing activities in students. This can be considered as **Hypothesis 6**. The results confirm the indirect partial and negative effects of cyberloafing behaviour on cyberloafing activities through psychological wellbeing (*z* = −2.366, *p* ≤ 0.05).

In the second mediation effect, we tested whether social media learning mediates the relationship between cyberloafing behaviour and cyberloafing activities in students. This can be considered as **Hypothesis 7**. The results confirm the indirect partial and negative effects of cyberloafing behaviour on cyberloafing activities through social media learning (*z* = −1.9754, *p* ≤ 0.05). Table 4 summarizes these results.

### 4.6. Hayes Process Macro in SPSS

Next, we utilized the Process Macro in SPSS to investigate the null hypothesis. The Process Macro provides different test statistics that explain the direct, indirect, and total effects, along with the total and partial effect sizes. Using Process Macro in SPSS, we performed a bootstrapping method to further examine the mediating effects.

First, we examined whether psychological wellbeing mediated the relationship between cyberloafing behaviour and cyberloafing activities. The regression analysis results show that cyberloafing behaviour significantly predicts psychological wellbeing (*b* = −0.179, *t* = −3.368, *p* < 0.001). Next, while controlling for psychological wellbeing, the results of the second regression analysis showed that cyberloafing behaviour was a significant predictor of cyberloafing activities (*b* = 0.311, *t* = 6.475, *p* < 0.05). The indirect effect results based on 5000 bootstrap samples show a significant indirect negative relationship between cyberloafing behaviour and cyberloafing activities mediated by psychological wellbeing (a × b = −0.034, Bootstrap CI95 = −0.068 and −0.008). The mediator, psychological wellbeing, accounted for approximately 12.23% of the total effect of cyberloafing activities [PM = (−0.034)/(0.278)]. Also, there was a statistically significant direct effect between cyberloafing behaviour and cyberloafing activities (*b* = 0.311, *t* = 6.475, *p* < 0.05). Therefore, this mediation analysis result also interprets that there is partial mediation. Partial mediation occurs in the case in which the path from the independent variable to the dependent variable is reduced in absolute size but is still different from zero when the mediator is introduced [106]. In other words, the independent variable (cyberloafing behaviour) has both direct and indirect effects on a dependent variable (cyberloafing activities). The direct effect is not mediated, whereas the indirect effect is transmitted through one mediator variable (psychological wellbeing). Table 5 displays the results of the mediation analysis.

Thereafter, we examined whether social media learning mediated the relationship between cyberloafing behaviour and cyberloafing activities. The regression analysis results show that cyberloafing behaviour was a significant predictor of social media learning (*b* = −0.121, *t* = −2.668, *p* < 0.05). Next, while controlling for social media learning, the results of the second regression analysis showed that cyberloafing behaviour was a significant predictor of cyberloafing activities (dependent variable (*b* = 0.302, *t* = 6.296, *p* < 0.05). The indirect effect results based on 5000 bootstrap samples show a significant indirect negative relationship between cyberloafing behaviour and cyberloafing activities mediated by social media learning (a × b =−0.024, Bootstrap CI95 = −0.052 and −0.003). The mediator, social media learning, accounted for approximately 8.633% of the total effect on cyberloafing activities [PM = (−0.024)/(0.278)]. Also, there was a statistically significant direct effect between cyberloafing behaviour and cyberloafing activities (*b* = 0.302, *t* = 6.296, *p* < 0.05). Therefore, this mediation analysis result interprets that there is partial mediation. In other words, the independent variable (cyberloafing behaviour) has both direct and indirect effects on a dependent variable (cyberloafing activities). The direct effect is not mediated, whereas the indirect effect is transmitted through one mediator variable (social media learning). Table 6 displays the results of the mediation analysis.

## 5. Discussion and Implications

The present study examined the mediating effects of psychological wellbeing and social media learning on the relationship between cyberloafing behaviour and cyberloafing activities of students in higher education. Cyberloafing behaviour positively relates to cyberloafing activity, as a negative attitude towards class can increase cyberloafing activities [10]. The results indicate that cyberloafing behaviour negatively influences students’ psychological wellbeing, which is in line with previous studies [107,108,109]. Inappropriate use of technology can result in higher depression, increased stress, and reduced psychological wellbeing [110,111]. Students who have access to technology in classrooms mostly use it for socializing, sharing, shopping, real-time updating, and accessing content online, which can hamper their academic performance [7,10]. It can be argued that reduced psychological wellbeing could be an outcome of a lack of motivation, lower performance, lower academic success, and decreased commitment due to increased use of the internet for the noneducational purpose during class time [31,41,112].

This study also demonstrates that psychological wellbeing is positively related to cyberloafing activities. When students are more satisfied and more confident, they will engage in cyberloafing activities to increase social connectedness, relax, and increase their knowledge [113,114]. Previous research supports a positive association between psychological wellbeing and cyberloafing activities. This relationship has been primarily studied in unidirectional ways, i.e., how cyberloafing activities affect wellbeing or how stress induces cyberloafing activities. Supporting the hypothesis, the results show that cyberloafing behaviour is positively related to cyberloafing activity. When students are using technology for noneducational purposes, the chances of engaging in any cyberloafing activities, like surfing the net, blogging, using social network sites, and watching online videos, will also increase. Given that cyberloafing behaviour negatively influences psychological wellbeing [108], student’s frustration, boredom, stress, and fatigue may increase. This result is in line with the conservation of resource theory when cyberloafing behaviour negatively affects psychological wellbeing; students are losing their personal resource. In order to cope with increased stress and fatigue, students may engage in cyberloafing activities, like gaming and watching a video, especially when students are interested in the course, which may act as a recovery mechanism [115]. Recent studies show that cyberloafing activities can act as a strategy to recover from boredom and stress [116].

The results show that cyberloafing behaviour negatively influences social media learning. Cyberloafing distracts students’ attentions away from class-related activities [40]. Students engage in cyberloafing behaviour to escape from class activities [117]. Such use of ICT in learning environments diverts the student and inhibits the motivation for indepth learning [112]. When students are not motivated, they show less interest in class activities and in comprehending the content. Rather than using technology for connecting with classmates and engaging in class discussions, students may use it for purposes irrelevant to class activities like surfing the internet and following social media. Students are multitasking due to their constant availability and fast responsiveness to different media, which has a negative impact on their attention, class discussion, and participation [45,118]. This result is in line with the research that demonstrates cyberloafing as a counterproductive behaviour in the context of students [119] and employees [120,121].

It was also found that social media learning does not affect cyberloafing activities. These results contradict previous research that shows a positive association between social media learning and cyberloafing activity [79]. It is believed that while using social media for learning, students may get distracted and get involved in multitasking, increasing their cyberloafing activities [33]. This may be because we measured social media learning from a perspective of students’ active participation in class discussions using social media.

The results of the mediation analysis were significant and negative for cyberloafing activities. A full mediation model was not supported, as the direct effect of cyberloafing behaviour was still significant. Psychological wellbeing and social media partially mediate the relationship between cyberloafing behaviour and activity. Accordingly, there are likely multiple factors that mediate the association between cyberloafing behaviour and activities. Other factors, like academic pressure, teacher’s support, engagement level, and subjective and descriptive norms [41,122], are also determinants of the buffering mediating mechanism. Students’ cyberloafing behaviour can negatively influence their psychological wellbeing and social media learning, which reduces their cyberloafing activities.

This study contributes to the cyberloafing literature in the following ways. The study focuses on the influence of cyberloafing within an educational setting. Given the widespread use of smartphones among students and the inevitability of technology integration in classrooms, it becomes crucial to assess their effects on students’ educational encounters and academic achievements. Further, this study considers cyberloafing as a multidimensional construct rather than a one directional construct. Moreover, previous investigations on cyberloafing in educational environments primarily concentrated on conventional measures of academic performance, like grade point averages (GPA). Nevertheless, since GPA alone does not provide a comprehensive understanding of students’ learning experiences, scholars have underscored the importance of exploring subjective and psychological learning outcomes. Therefore, deviating from prior studies that predominantly centred on traditional learning outcomes such as grades, this research emphasizes the examination of psychological wellbeing and the role of social media as a mechanism connecting cyberloafing behaviour with cyberloafing activities in the context of learning. Such a study may help both academics and teachers comprehend the fundamental causes of technology misuse in the classroom.

## 6. Practical Implications of the Study

The current study provides insightful clues on how cyberloafing behaviours have an impact on psychological wellbeing and learning through social media in students. The prevalence rates of cyberloafing are accelerating across university students with the degree of access to emerging mobile technologies. Even though it is difficult to employ technical countermeasures in educational settings, it has become a need of the hour for course facilitators and instructors to create such solutions to a certain extent, as it is not possible to completely eliminate or limit the use of emerging ICTs. It would be very useful to design proper awareness-raising interventions to mitigate further unregulated and counterproductive use of emerging information and communication technologies.

Moreover, it is important to take feedback from students and understand their perceptions regarding the quality, nature, and pace of instructional activities to understand the reasons for counterproductive internet usage behaviours. This would not only reduce cyberloafing activities within class but it would also give the course facilitators and instructors an understanding of how to monitor and enhance the overall effectiveness of the course. In turn, this would also help students to manage their psychological wellbeing and improve their learning effectiveness in class.

Lastly, the course facilitators should understand that if students feel they are heard and respected by their instructors and peers, they will be less likely to engage in cyberloafing during class hours and more likely to pay attention to the course contents. It is the responsibility of the instructors to design rules for using the internet during classes. The rules should be flexible enough to allow students to take advantage of cyberloafing to take short breaks intermittently, relax, and eliminate their mental stress loads. Foster [123], for example, ran an experiment in which she asked her pupils to turn off their phones and place them in a basket in front of the class. Students debated the pros and cons of this practice towards the conclusion of the class. Analysis revealed that the students performed well in the lesson and reported appreciating it more [123]. Such an approach is more inclusive and less invasive, and students appear to like it. The universities would also benefit from creating an effective training workshop or seminar such as “ethics in computing curriculum” that highlights the counterproductive outcomes of cyberloafing and makes students aware of ineffective and inefficient use of technology during classes [124].

## 7. Limitations and Future Research Directions

The current study also has a few limitations. First, we followed a cross-sectional design and collected data at only one time point. It would be interesting to collect data at different time points (time-lagged fashion) during the semester to better understand the temporal effect of different variables. Moreover, data was collected using convenience sampling rather than random sampling, as their use is typical in educational research, where constraints such as time, money, and resources make random sampling infeasible [125]. However, future studies need to incorporate different data-collection methods so that the study findings can be generalized.

Second, even though measures were taken to reduce socially desirable responses, it is likely that self-reported cyberloafing behaviour may not fully represent actual behaviour, and students might have responded in a socially desirable manner as cyberloafing is a sensitive topic [39]. Lastly, we used the same five-point Likert scale to measure all the variables in the study. This could cause a lack of meticulous response and method bias, thus restraining the accuracy of our findings. Further research could follow Podsakoff et al. [98] and be careful in using multiple scales to measure the variables. It would also be beneficial to use a fusion of different research approaches to examine the interrelationships between the variables more comprehensively.

In this study, we have considered shopping, searching for social support, expression of opinion, gaming, and social network extension as cyberloafing activities. Future research may consider other cyberloafing activities like blogging, accessing online content, and sharing information. Lastly, this study was carried out in the Asian region and the results might vary if the study is replicated in other geographies. Moreover, the sample is from a single university in India, which may limit the applicability of the findings to other cultural contexts. Further studies could do a comparative study to understand cyberloafing behaviour in different regions and other cultural contexts.

## 8. Conclusions

From an educational standpoint, numerous aspects are carried out nowadays at educational institutions in an effort to benefit from information and communication technologies. As a result, ICT has become an integral part of education. Students can benefit from internet information sources in their learning processes by using the wired and wireless networks that these institutes are equipped with. Despite the numerous benefits that these technologies have brought to the learning–teaching processes, the inappropriate, excessive, and uncontrolled use of these technologies by learners has resulted in a number of issues, including cyberloafing. Given the growing incidence of internet use among students, research into cyberloafing in educational contexts is critical. We assessed the relationship between cyberloafing behaviour and cyberloafing activities and the mediating role of social media learning among students. Past research on cyberloafing concentrates on the positive and negative impacts on students’ performance and wellbeing. The present study broadened the focus of cyberloafing behaviour and provided a model that explains how cyberloafing behaviour can reduce social media learning and wellbeing, and increase cyberloafing activities, which is the first of its kind. The findings confirm prior research and further the body of work already in existence.

## Figures and Tables

**Table 1:** Sociodemographic of the respondents.

Characteristic	Count *	Percent
Gender		
Male	124	51.7
Female	116	48.3
Age		
17–20	215	89.6
20 and above	25	10.4
Education department		
Engineering	142	59.2
Law	66	27.5
Management	32	13.3

* *n* = 240.

**Table 2:** Means, standard deviations, and correlations.

S No.	Variables	Mean	S.D.	1	2	3	4
1	Cyberloafing activities	2.3181	0.7693				
2	Cyberloafing behaviour	2.4781	0.9736	0.351 **			
3	Psychological wellbeing	3.4475	0.8158	0.116	−0.213 **		
4	Social media learning	3.6008	0.6907	0.112	−0.170 **	0.245 **	

Notes: *n* = 240; ** *p* < 0.01 (two-tailed test).

**Table 3:** A summary of model-fit indices.

Model Test	χ^2^	df	SRMR	CFI	GFI	RMSEA
**Independence model**	1894.701	190				
**Measurement model**	266.252	160	0.0683	0.938	0.904	0.053
**Hypothesized model**	276.311	161	0.0803	0.932	0.900	0.055

Note: χ^2^ = Chi-square; df = Degrees of freedom; SRMR = Standardized Root Mean Square Residual; CFI = Comparative fit index; GFI = Goodness-of-fit statistic; RMSEA = Root mean square error of approximation.

**Table 4:** Hypothesis testing results for the research model.

Hypotheses	Relationships	Standardized Regression Coefficients	*t*-Values	*p*-Values	Hypotheses Results
**H1**	cyberloafing behaviour → psychological wellbeing	190	−3.432	<0.01	Supported
**H2**	psychological wellbeing → cyberloafing activities	160	1.981	<0.048	Partially supported
**H3**	cyberloafing behaviour → cyberloafing activities	161	5.370	<0.01	Supported
**H4**	cyberloafing behaviour → social media learning	52	−2.521	<0.012	Supported
**H5**	social media learning → cyberloafing activities	52	0.938	0.348	Not supported

**Table 5:** Sobel test results.

			Sobel Test	
			*t*-Statistic	*p*
Cyberloafing behaviour	Psychological wellbeing	Cyberloafing activities	−2.366	0.05
Cyberloafing behaviour	Social media learning	Cyberloafing activities	−1.9754	0.05

**Table 6:** (**a**) Mediation analysis results for CLB → PSW → CLA. (**b**) Mediation analysis results for CLB → SML → CLA.

(**a**)						
**Variable/Effect**	***b***	***SE***	***t***	***p***	***95% Confidence interval***	
CLB → CLA	0.311	0.048	6.475	0.000	0.217	0.406
CLB → PSW	−0.179	0.053	−3.368	0.001	−0.283	−0.074
CLB → PSW → CLA	0.189	0.057	3.288	0.001	0.076	0.302
***Effects***						
Direct	0.311	0.048	6.475	0.000	0.217	0.406
Indirect ×	−0.034	0.015			−0.068	−0.008
Total	0.278	0.048	5.792	0.000	0.183	0.372
(**b**)						
**Variable/Effect**	***b***	***SE***	***t***	***p***	***95% Confidence interval***	
CLB → CLA	0.302	0.048	6.296	0.000	0.207	0.396
CLB → SML	−0.121	0.045	−2.668	0.008	−0.210	−0.032
CLB → SML → CLA	0.198	0.068	2.926	0.004	0.065	0.331
***Effects***						
Direct	0.302	0.048	6.296	0.000	0.207	0.396
Indirect ×	−0.024	0.013			−0.052	−0.003
Total	0.278	0.048	5.792	0.000	0.183	0.372

Note: Based on 5000 bootstrap samples.

## Data Availability

The data will be made available on request.

## Appendix A

**Table A1:** Measurement items.

Variables and Items
**Cyberloafing activities**
1. Shop online.
2. Search for social support.
3. Express my opinion—Twitter/LinkedIn.
4. Save a game.
5. Extend social network.
6. Play an online game.
**Cyberloafing behaviour**
1. Avoid school tasks.
2. Avoid thinking of work tasks.
3. Postpone work tasks.
4. Acquire abilities.
**Psychological wellbeing**
1. I have been thinking clearly.
2. I have been feeling good about myself.
3. I have been feeling confident.
4. I have been able to make up my own mind about things.
5. I have been feeling cheerful.
**Social media learning**
1. I feel a sense of community learning becomes interactive.
2. Posting questions to my classmates/friends helps me understand my readings better.
3. I am able to get faster feedback from my peers.
4. I am able to get faster feedback from my instructor.
5. I am able to communicate effectively.
